# Highly sensitive hot electron bolometer based on disordered graphene

**DOI:** 10.1038/srep03533

**Published:** 2013-12-18

**Authors:** Qi Han, Teng Gao, Rui Zhang, Yi Chen, Jianhui Chen, Gerui Liu, Yanfeng Zhang, Zhongfan Liu, Xiaosong Wu, Dapeng Yu

**Affiliations:** 1 State Key Laboratory for Artificial Microstructure and Mesoscopic Physics, Peking University, Beijing 100871, P. R. China; 2 Collaborative Innovation Center of Quantum Matter, Beijing 100871, P. R. China; 3 College of Chemical and Molecular Engineering, Peking University, Beijing 100871, P. R. China

## Abstract

A bolometer is a device that makes an electrical resistive response to the electromagnetic radiation resulted from a raise of temperature due to heating. The combination of the extremely weak electron-phonon interactions along with its small electron heat capacity makes graphene an ideal material for applications in ultra-fast and sensitive hot electron bolometer. However, a major issue is that the resistance of pristine graphene weakly depends on the electronic temperature. We propose using disordered graphene to obtain a strongly temperature dependent resistance. The measured electrical responsivity of the disordered graphene bolometer reaches 6 × 10^6^ V/W at 1.5 K, corresponding to an optical responsivity of 1.6 × 10^5^ V/W. The deduced electrical noise equivalent power is 1.2 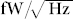, corresponding to the optical noise equivalent power of 44 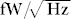. The minimal device structure and no requirement for high mobility graphene make a step forward towards the applications of graphene hot electron bolometers.

One of many astonishing properties of graphene is its weak electron-phonon (e-p) coupling. In normal conductor, e-p scattering quickly dominates as the temperature increases, resulting in a diminishing carrier mobility. In contrast, e-p scattering in graphene is negligible even at room temperature^1^,^2^. Consequently, graphene has so far the highest mobility at room temperature among all the other materials^3^,^4^,^5^. Because of its high mobility, applications in high speed transistors^6^,^7^,^8^ and in highly conductive interconnects have been foreseen. Recently, significant interest has grown in using graphene as a photodetector by exploiting its weak e-p interactions^9^,^10^,^11^,^12^,^13^,^14^,^15^,^16^. One type of such detectors is known as hot electron bolometer (HEB). Potentially, graphene HEB is very sensitive and extremely fast at the same time^17^, due to its small electron heat capacity and the low thermal conductance between the electron and phonon gases. Several attempts have been made in realizing graphene HEBs and the main goal is to obtain a large temperature coefficient for the resistance of graphene. For example, an energy gap was introduced either by applying a strong magnetic field to form Landau levels^11^, by a dual-gated bilayer graphene^13^ or alternatively by using a superconducting tunnel junction^14^. In a different approach, the electronic temperature was measured by means of the noise thermometry instead of the resistance^18^,^19^,^20^. However, a simple resistive readout would be preferred in practice. Furthermore, it would be highly desired if a graphene HEB can be made without acquiring high quality graphene films and dedicated micro-fabrication process and can operate without a magnetic field.

This present work demonstrates a new approach for implementing a graphene HEB. The main key is to drive the electronic system into the strong localization regime by adding disorder. Note that disorder here in general denotes the scattering potentials of various forms, such as lattice defects, charge impurities, etc. The divergence of the resistance at low temperature will allow for sensitive temperature measurement. The high resistance, together with an as-grown Boron Nitride (BN) tunneling barrier between graphene and electrical contacts, also reduces thermal dissipation out of the electron gas via diffusion. The bolometer fabricated with such method is found highly sensitive and with a very low background noise. Moreover, this approach does not require graphene with high mobility. The device structure with merely a graphene bar in contact with two electrodes can be easily fabricated with standard photo-lithography technique and extended into a 2D detector array.

## Results

**Figure 1:**
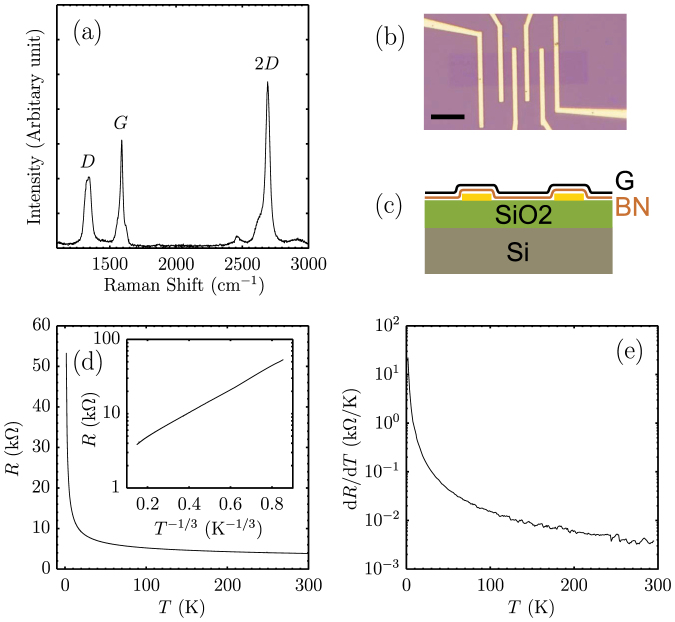
Disordered graphene film on BN. (a) Raman spectrum of disordered graphene on BN. The amplitude of the disorder peak (*D* peak) is over half of that of the *G* peak. (b) Optical image of a device. The scale bar represents 5 *μ*m. (c) Diagram of a device, showing a graphene film which is separated from the electrodes by a BN film. (d) The temperature dependence of the resistance for a 2.5 *μ*m long and 5 *μ*m wide graphene ribbon. Inset, the resistance is plotted against *T*^−1/3^. Note that the *y* axis is in a logarithmic scale. (e) d*R*/d*T* as a function of temperature.

The samples used in this study were Graphene/BN bilayer grown by CVD method^21^. In this method, monolayer BN is first grown on copper foil, followed by growth of graphene on top. Depending on the growth condition, the quality of graphene films can be easily controlled and finely tuned. In order to obtain the defective graphene films, a high carbon precursor flux and a low growth temperature were employed in promoting dense nucleation^22^. Films grown by this means exhibit significant defects, as evidenced by a strong *D* peak in the Raman spectrum in Fig. 1(a)^23^,^24^.

Our bolometer devices consist of a 5 *μ*m wide BN/Graphene ribbon on the metal electrodes, see Fig. 1(b) and (c). The graphene film is separated from the electrodes by the BN layer, which would be acting as a tunneling barrier. Its role is to increase the electrical contact resistance, hence the thermal resistance. The result is better thermal isolation. An estimation and discussion of the thermal resistance will be given later. The resistance of such graphene films increases with the decreasing temperature and then diverges at low temperature, shown in Fig. 1(d). We plot the resistance *R* against *T*^−1/3^ in a semilog scale, where a linear dependence spans from the lowest temperature of 1.5 K to 300 K. The data suggest that electrons are strongly localized and the transport is dominated by the so-called variable range hopping (VRH)^25^. For VRH, the resistivity *ρ*(*T*) ∝ exp[(*T*_0_/*T*)^1/3^]. Here, *T*_0_ is a characteristic temperature, *T*_0_ = 12/*πk*_B_*g*(*E*_F_)*ξ*^2^, where *k*_B_ is the Boltzmann constant, *g*(*E*_F_) the density of states at the Fermi level *E*_F_, *ξ* the localization length. We have performed a linear fit for the ln *R* versus *T*^−1/3^ plot. The slope of the fit yields a localization length *ξ* of 50 nm. Both the Raman results and the transport data indicate that the graphene film is disordered. The sharp increase of the resistance at low temperature due to localization is the key ingredient of our bolometer, as the responsivity of our bolometer is proportional to the slope of the *R* – *T* curve, d*R*/d*T*. Fig. 1(e) shows d*R*/d*T* as a function of temperature. At 2 K, d*R*/d*T* reaches 22 kΩ/K. Moreover, a high electrical resistance means a high thermal resistance, which prevents heat from dissipation via diffusion and also helps the responsivity.

**Figure 2:**
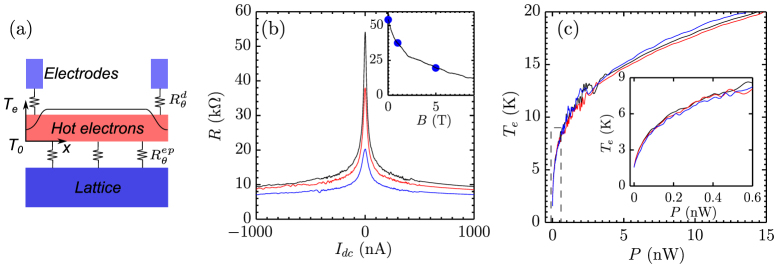
Hot electrons generated by Joule heating in disordered graphene bolometer. (a) A schematic for heat dissipation in a device. The graphene lattice and the electrodes are assumed at the bath temperature *T*_0_^13^. Two heat dissipation paths, diffusion and e-p scattering are denoted by 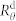 and 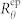, respectively. The longitudinal temperature distribution *T*_e_(*x*) is drawn in solid line, when 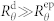. The temperature is relatively uniform, while it deviates above the electrodes due to diffusion. (b) Strong nonlinear resistances as a function of the bias current *I*_dc_. Black, red, blue lines are data at *B* = 0, 1, 5 T, respectively. The magnetoresistance is shown in the inset. The solid circles denote the fields in which the bias dependence were measured. To minimize heating by the a.c. current, 0.5 nA a.c. current was used at low bias, while 10 nA was applied at bias *I*_dc_ > 500 nA to enhance the signal-to-noise ratio. (c) Scaling of the bias dependence of *R* in (b). *T*_e_ is obtained from *R* according to *R* – *T*, while *P* = *I* × *V*. The inset is a zoom-in view of the area enclosed by the dashed line in the main panel, which shows that the scaling is very good at low bias, too.

To see how the bolometer responses to heating, we apply a d.c. bias current *I*_dc_ to generate heat in the electron gas. As shown in Fig. 2(b), the differential resistance drops sharply with the bias current. One possible source for the nonlinearity is the field dependence of the resistance in the variable range hopping regime. It originates from field-assisted hopping, which is described by *V*/*I* = *R*_0_ exp(−*eEl*/*k_B_T*) when *eEl*/*k_B_T* < 1^26^. Here *R*_0_ is the zero bias resistance, *E* the electric field, *e* the electron charge and *l* a fraction of the hopping distance *r_m_*, *i.e.*, *l* = 0.18*r_m_*. Since *r_m_* = *ξ*(*T*_0_/*T*)^1/3^, we have *l* = 29 nm at 1.57 K. A fit of the data to an exponential field dependence is very poor, see Fig. S1 and the change of the resistance due to field-assisted hopping is substantially less than the experiment, about one fourth of the experimental value at a bias voltage of 3 mV. Moreover, as the electronic temperature increases due to Joule heating, field-assisted hopping exponentially diminishes. Thus, it is unlikely the origin of the nonlinearity.

We argue in the following that the nonlinear resistance is the consequence of hot electrons created by Joule heating, consistent with previous experiments^13^,^27^,^28^. Considering the heat dissipation of the electron gas in the device, there are two possible pathways, by diffusion to electrodes or by transfer to the graphene lattice via the e-p scattering. The two pathways are schematically drawn in Fig. 2(a). The heat resistance due to diffusion, 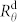, will give rise to a temperature gradient along the graphene ribbon^27^, while the heat resistance due to the e-p scattering, 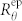, dissipates heat uniformly. When the e-p pathway is dominant, the electron temperature *T*_e_ will be determined by the strength of the e-p scattering and nearly uniform along the sample. We now show that this is indeed true in our devices. For a uniform temperature, *T*_e_ at different biases can be obtained from the resistance *R* based on the *R* – *T* curve. We have measured *R* – *I*_dc_ in magnetic fields of 0, 1, 5 T (Fig. 2(b)). Although the resistances are very different in three fields, after converting *R* to *T*_e_ and *I*_dc_ to Joule power *P*, all data collapse onto a single *T*_e_ – *P* curve, shown in Fig. 2(c). The scaling works surprisingly well for the whole range of the bias. The same scaling was also found in other devices (see Supplementary Fig. S3 online). It is a strong evidence for the proposed thermal model in Fig. 2(a) and Joule heating being the origin of the nonlinear resistance. More confidence in the model can be gained by analyzing the thermal resistance for the two pathways. The contact resistances are about 20 kΩ, which would yield a total contact thermal resistance of 273 K/nW for two contacts, according to the Wiedemann-Franz law. The total thermal resistance of the device is *R_θ_* = d*T*_e_/d*P* ≈ 58 K/nW when *T*_e_ ≈ *T*_0_, much less than the contact thermal resistance. As *T*_e_ increases, *R_θ_* sharply reduces. Therefore, the dissipation is mainly dominated by e-p scattering.

**Figure 3:**
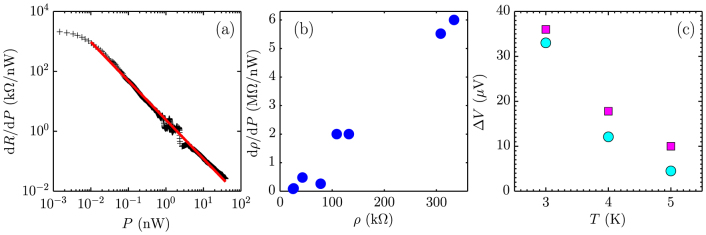
Responsivity of disordered graphene bolometer. (a) the slope of the resistance versus the Joule power, d*R*/d*P*, at *T* = 1.5 K. The red line is a linear fit, which gives d*R*/d*P* ∝ *P*^−2.3^. (b) The dependence of the resistive responsivity, d*ρ*/d*P*, on disorder, measured by the sheet resistance *ρ*. (c) The response of the graphene bolometer to Joule heating (solid cyan circle) and radiation heating (solid magenta square) at a power of 40 pW. The rough estimation of the radiation power can be found in the Supplementary online.

It becomes clear that the electron gas in our disordered graphene device is heated by the bias current. The electron temperature increases nearly uniformly along the device, leading to a decreasing resistance. We can calculate the electrical responsivity of the bolometer, which is the ratio of the voltage response to the Joule power^29^. In Fig. 3(a), the slope of the resistance *R* versus *P*, d*R*/d*P*, is plotted against *P*. The resistance sensitively responses to *P*, following a d*R*/d*P* ∝ *P*^−2.3^ behavior. When *P* < 0.01 nW, d*R*/d*P* is over 1 MΩ/nW. Using an a.c. excitation current of 2 nA, at which no appreciable heating occurs, we obtain a responsivity of 2 × 10^6^ V/W at 1.5 K. The responsivity can be greatly improved if the temperature is to be further decreased, as the VRH resistance diverges at low temperature.

The responsivity of a graphene HEB depends mainly on two factors. One is the e-p coupling strength, which determines the amplitude of the temperature increase in response to a certain power input. The other is the voltage response to the temperature change, which is the sensitivity of graphene as a thermometer. In Fig. 1(e), it has been shown that disordered graphene exhibits a strong temperature coefficient. The temperature coefficient stems from localization of electrons by disorder and follows the VRH behavior. By varying the degree of disorder, the coefficient can be significantly tuned. We have measured devices of graphene films with sheet resistances *ρ* ranged from 30 to 350 kΩ. The change of the resistance in response to the power, d*ρ*/d*P*, at *P* = 10 pW is plotted as a function of *ρ* in Fig. 3(b). It linearly follows *ρ*. The highest value in our experiment reaches 6 MΩ/nW. The corresponding responsivity of the device, which is 2.5 *μ*m long and 5 *μ*m wide, is 6 × 10^6^ V/W at an excitation current of 2 nA.

To test the actual bolometric response of the device, we employed a red LED to illuminate the device. The LED emits light with a peak light wavelength of 650 nm. Since the whole device is under uniform and constant illumination, photovoltaic and photothermoelectric signals will not be picked up by the lock-in (See Method). Considering a multilayer structure, *i.e.*, graphene/BN/SiO_2_/Si, the transfer matrix of the structure is deduced^30^ and the absorbed power by graphene is estimated to be 40 pW (2.7% absorptance, see the Supplementary online). With an excitation current of 1 nA, the voltage responses Δ*V* at different temperatures are plotted in Fig. 3(c). As a comparison, the responses to the same amount of Joule power are also plotted. We find a good qualitative agreement between two responses, which justifies our characterization of the bolometer using Joule heating^13^.

## Discussion

**Figure 4:**
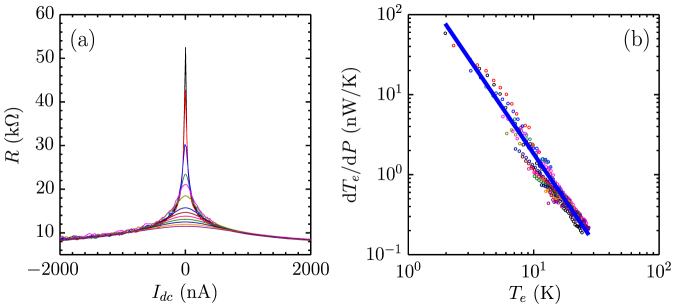
Thermal resistance of disordered graphene bolometer. (a) Bias dependence of the differential resistance of the device at 1.57, 2, 3, 4, 5, 6, 7, 8, 9, 10, 11, 12 K. (b) d*T*_e_/d*P* as a function of *T*_e_. The solid blue line is a linear fit, which gives 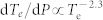.

We have shown that the thermal dissipation pathway is mainly dominated by e-p scattering. Therefore, the thermal resistance due to e-p scattering can be computed by 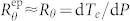. Fig. 4(a) shows the bias dependence of resistance at different temperatures. We can convert the *R* – *V*_dc_ plot into a *T*_e_ – *P* plot and then numerically compute the derivative d*T*_e_/d*P*, as plotted in Fig. 4(b). log 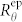 displays a linear dependence on log *T*_e_, which is expected by theoretical work on e-p scattering in graphene. By fitting the linearity, we have 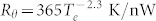. It has been predicted that the thermal resistance due to e-p scattering follows 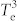 when 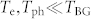 and becomes 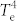 when 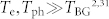
2,31. Here, *T*_ph_ is the phonon(lattice) temperature, while *T*_BG_ is Bloch-Grüneisen temperature, defined as *k*_B_*T*_BG_ = 2*k*_F_*v*_ph_, where *k*_F_ is the Fermi wave vector, *v*_ph_ the speed of sound^5^. For our sample with a measured Fermi level of 0.23 eV and the speed of sound *v*_ph_ = 2 × 10^4^ m/s, *T*_BG_ ≈ 102 K, much higher than *T*_e_ and *T*_ph_. Considering a deformation potential *D* = 18 eV^32^ and Fermi velocity *v*_F_ = 10^6^ m/s, we find that for a graphene film with an area of 12.5 *μ*m^2^, 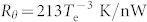
31. Note that these theories assume unscreened deformation potential and clean limit. Our result is in fact in good agreement with the theoretical prediction. We now turn to the role of disorders on e-p coupling. It has been proposed that disorder-assisted cooling pathway (supercollison) can be substantial for disordered graphene as it contributes to the energy loss rate besides conventional electron-phonon scattering^33^. Our experiment gives a larger thermal resistance comparing to the theoretical value, suggesting absence of supercollision. The result shown here is consistent with previous experimental studies^13^,^19^. Recently experiments have suggested suppercollision in graphene when 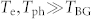
34,35, while the normal e-p scattering dominates when 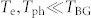, which does not contradict our results. The effect of disorder on e-p coupling can be considered an intriguing topic and it would be interesting to study. However, it is out of the scope in this work.

Having known the value of the thermal resistance, the response time *τ* of the bolometer can now be estimated by *ρ* ≈ *R_θ_C_e_*, where *C_e_* is the electronic heat capacity. 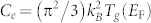 when 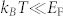. At 1.5 K, the electronic heat capacity of our sample is about 2.4 × 10^−20^ J/K, resulting in *τ* ≈ 1.4 nS. Apparently, the intrinsic speed of graphene bolometer is extremely fast. However, in practical application, the actual response time is also limited by the RC time constant of the whole circuit, which is *τ* = *RC*. Here, *R* and *C* are the circuit resistance and capacitance, respectively. Take the sample above as an example. Considering the total area of four metal electrode pads and the graphene sample, the capacitance to the back gate is estimated to be 7 pF. The total capacitance of our measurement cables is roughly about 250 pF, while the total resistance, including the sample resistance and two contact resistances, is 94 kΩ. We then have *τ* ≈ 24 *μ*s. Therefore, it is important to minimize the parasitic capacitance of the bolometer circuit and the resistance of graphene in order to approach the intrinsic speed of graphene HEBs.

Besides the responsivity, noise equivalent power (NEP) is a key factor that determines the performance of a bolometer. There are three noise sources, the measurement circuit noise, the thermal fluctuations and the Johnson noise^14^. The first one is extrinsic and depends on how the voltage measurement circuit is implemented. We now derive the latter two which are intrinsic to the device^13^. We have already obtained the thermal resistance *R_θ_* ≈ 58 K/nW at 1.5 K. Thus, NEP by the thermal fluctuations is 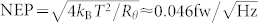. The Johnson noise is 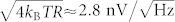 for *R* = 54 kΩ plus the total contact resistance of 40 kΩ. The corresponding NEP is the noise divided by the responsivity, 
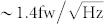
. The NEP value on the device with the highest responsivity is indicated as 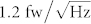.

When estimating the responsivity and NEP of our devices, we have used the absorbed power. Although this gives the ultimate performance of the device, it is useful to discuss the optical responsivity and NEP, estimated by taking the incident radiant power as the input. Taking into account the 2.7% absorptance of the device, we get an optical responsivity, 6 × 10^6^ × 0.027 ≈ 1.6 × 10^5^ V/W and a NEP of 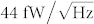. Therefore, it is important to increase the absorptance in order to obtain a better performance. This can be accomplished by using multilayer graphene, surface plasmonic enhancement^36^ or a microcavity^37^, although it is important to remember that the latter two introduce wavelength selectivity.

We now discuss the role of the BN layer. As we have explained, it increases the contact resistance, therefore reduces thermal dissipation by diffusion. As a result, the temperature is uniform along the device, which allows for straightforward derivation of the electron temperature and the thermal conductance and analysis of e-p scattering. However, the temperature uniformity is not a requirement for our bolometer, although it is helpful to gain the responsivity by suppression of heat dissipation into contacts. In addition to increasing the contact resistance, it can be achieved by enhancing the graphene resistivity or by modifying the device geometry, *e.g.*, the ratio of the device area to the contact area. In fact, for disordered graphene, the resistivity is relatively high so that the heat dissipation through diffusion can be substantially reduced. As seen in the Supplementary Fig. S5 online, the devices made of disordered graphene film without the BN layer has shown a strong nonlinear resistance similar to the BN/graphene devices.

**Figure 5:**
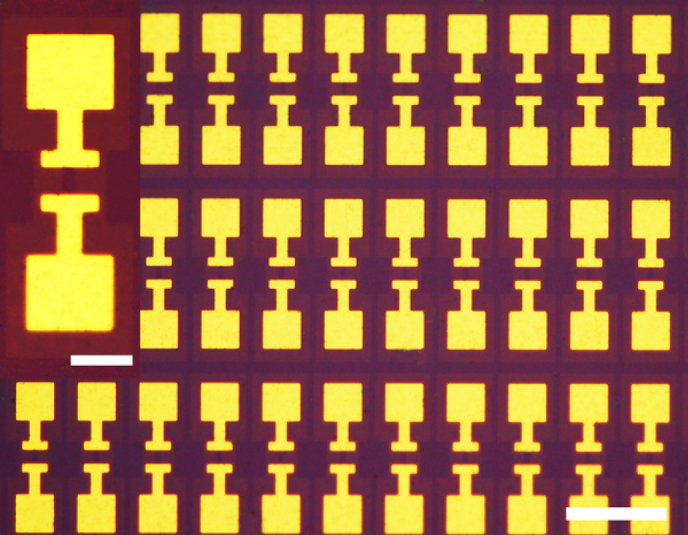
Optical images of an array of HEB structures. Golden areas are Au electrodes. The dark rectangle between two electrodes is graphene/BN. These devices are in a two-terminal configuration, on which no electrical measurement has been done on them. The scale bar in the main panel represents 40 *μ*m, while it is 10 *μ*m in the inset.

The basic structure of a disordered graphene HEB is comprised of only a graphene ribbon and two electrodes. It can be easily expanded to a detector array using conventional photo-lithography technique. In Fig. 5, we demonstrate the fabrication of an 2D array of HEB structures on a SiO_2_/Si substrate. Similar 2D array is the basic structure for 2D bolometric imaging, where each element works independently to provide a pixel. The graphene ribbon is 5 *μ*m long and 2 *μ*m wide. The fabrication procedure is similar to the one for making an individual device (see the Method). Although the e-beam lithography was used for this demonstration, photo-lithography is well suited for fabrication of the array. Note that the structure of the detector array has not been optimized, *i.e.*, the optical fill-factor is very low. We want to point out that the simplicity of the device structure and the aspect ratio dependent responsivity and resistance, offer significant flexibility in device design to meet applications.

Compared with previous approaches, *e.g.*, SdH oscillations, superconducting tunnel junctions and dual-gated bilayer graphene, disordered graphene provides a rather simple, yet effective scheme to implement a graphene HEB. The bolometer has an electrical responsivity of 6 × 10^6^ V/W, over an order of magnitude higher than other approaches^13^,^14^. When considering the light absorptance of graphene devices, the optical responsivity is estimated to be 1.6 × 10^5^ V/W. This value is much higher than 1 × 10^3^ V/W for the dual-gated bilayer graphene device^13^, as our device does not require a metal top gate, which substantially reduces the absorptance. The derived value of NEP is 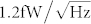 (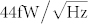 optical NEP), also an order of magnitude lower than the previous result^13^. It does not require high mobility graphene films and dedicated micro-fabrication. The device characteristics, such as resistance, responsivity, can be tuned by adjusting the degree of disorder. It works at liquid Helium temperature and can potentially work at a higher temperature if disorder is enhanced. It is worth to note that multilayer can increase the absorptance and decrease the device resistance. Such a multilayer structure can be constructed by simple stacking in our case, while it is difficult, if not impossible, to achieve in other approaches.

The responsivity of disordered graphene HEB rivals Si bolometers and composite bolometers^29^. The NEP is much lower than that of Si bolometer, 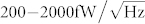, but it is still orders of magnitude higher than the state-of-art superconducting edge transition bolometer operating at 40 mK^38^. However, we expect these parameters to have great improvement when the temperature is lowered. The most important advantage of graphene HEB is that they are unprecedentedly fast^13^,^14^, a few nanoseconds compared with a few milliseconds for others.

## Methods

Bilayer structure of defective graphene/*h*-BN samples in this work were synthesized via low pressure chemical vapor deposition on 25 um Cu foils (purchased from Alfa Aesar)^21^. At the first stage, monolayer *h*-BN was initially grown on Cu foil at 1000°C, using ammonia borane (NH_3_BH_3_, purchased from Sigma-Aldrich) as the precursor^39^. Afterwards, direct growth of graphene on *h*-BN was achieved by introducing carbon sources into the system. Depending on the graphene growth parameters, *i.e.*, growth temperature and carbon source inlet flow rate, the quality of graphene can be controlled. In order to obtain defective graphene, low temperature (800°C) and high carbon source flux were chosen to increase densities of defects and nucleation in graphene film. The bolometer devices were fabricated by e-beam lithography. 20 nm Ti/50 nm Au electrodes were first deposited on Si/SiO_2_ (300 nm) substrates. The graphene/BN films were then transferred onto the substrate^40^. After that, the films were patterned into 5 microns wide ribbons. Four-probe electrical measurements were carried out using an a.c. lock-in method. Measurements on the bolometric response of devices were made by illuminating the device with a red LED which was placed over the sample.

## Author Contributions

X.S.W. conceived the experiment. X.S.W. and D.P.Y. supervised the experiment. Q.H. fabricated devices and performed low temperature measurements. T.G. synthesized BN/graphene samples. Y.F.Z. and Z.F.L. supervised sample synthesis. X.S.W., Q.H., Y.C. and G.R.L. wrote the manuscript. X.S.W., Y.C. and G.R.L. performed data analysis. R.Z. and J.H.C. helped with the experiment. All authors participated in data discussion and reviewed the manuscript.

## Supplementary Material

Supplementary Information Supplementary Info
