# Supplementary material for: Highly sensitive hot electron bolometer based on disordered graphene
Source: Sci Rep. 2013 Dec 18;3:3533. doi: 10.1038/srep03533 (PMC3866633; doi:10.1038/srep03533)
Supplement: Supplementary Information — Supplementary Info [file srep03533-s1.pdf]

Highly sensitive hot electron bolometer based on disordered graphene

Qi Han, Teng Gao, Rui Zhang, Yi Chen, Jianhui Chen, Gerui Liu, Yanfeng Zhang, Zhongfan Liu, Xiaosong Wu, Dapeng Yu

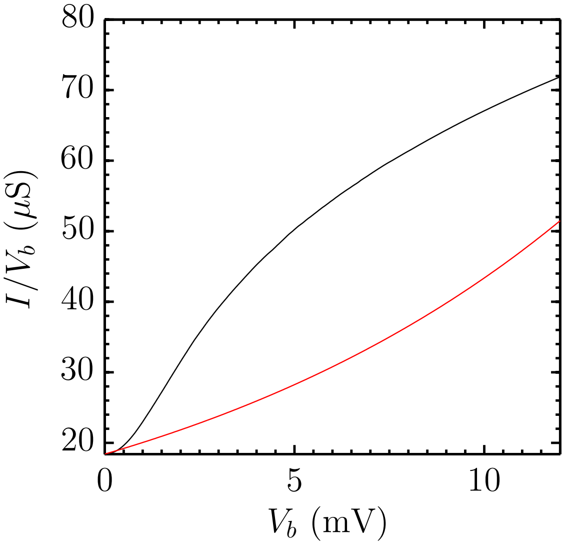

Fig. S1 Nonlinear IV characteristic of disordered graphene HEB. Black line, experiment data. Red line, simulation assuming field-assisted variable range hopping.

Disordered graphene bolometer displays a strong nonlinear resistance. We compare the data with field-assisted hopping, which predicts an exponential increase of the conductance, $I/V = G_0 \exp(-eEl/k_BT)$. Here G_0 is the zero bias conductance, e the electron charge, k_B the Boltzmann constant, l is a fraction of the hopping distance r_m. l is estimated as 29 nm, as seen in the main text. From the data, G_0 is 18.4 μS. Note that the length of the device is 2.5 μm. We obtain the field-assisted hopping conductance, as plotted in Fig. S1. The simulation not only shares little similarity with the experiment curve, but is way off from it. Moreover, since the contribution from field-assisted hopping decreases exponentially with temperature, a slight increase of the electronic temperature due to Joule heating will strongly reduce the contribution. Therefore, we exclude field-assisted hopping as the origin of the nonlinear resistance.

Figure S2(a) shows the bias dependence of resistance for a 5 μm wide and 2.5 μm long

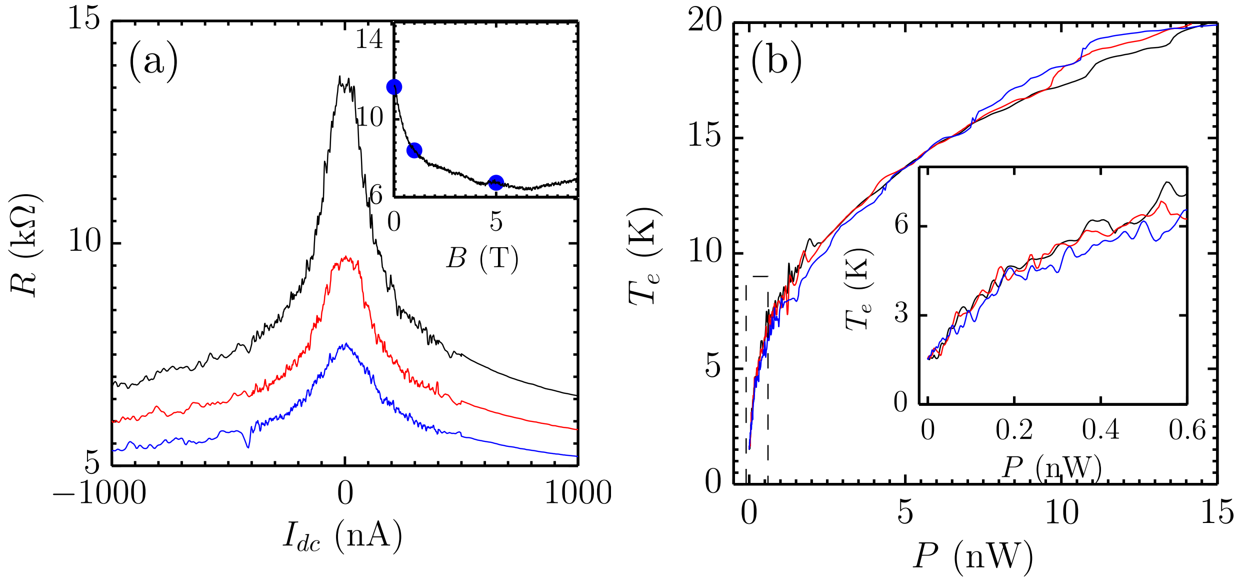

Fig. S2 Scaling of the bias dependence of resistance in disordered graphene. (a)Nonlinear resistances in three magnetic fields, B=0, 1, 5 T, represented by black, red, blue lines, respectively. The magnetoresistance is shown in the inset. The blue dots mark the fields in which the bias dependence of resistance were measured. To minimize the heating by the a.c. current, 0.5 nA was used at low bias, while 10 nA was applied at bias $I_{\rm dc} > 500$ nA to enhance the signal-to-noise ratio. (b)Electronic temperature $T_{\rm e}$, calculated from the bias dependence of resistance in (a), versus Joule power P in three magnetic fields. The inset is a zoom-in view of the area enclosed by the dashed line in the main panel, which shows that the scaling of three nonlinear resistance curves is very good at low bias, too.

device, which is less disordered compared with the one presented in the main text. The dependence of the resistance were measured at B=0, 1, 5 T. As discussed in the main text, the nonlinear resistance is due to Joule heating. If the major heat dissipation channel is through e-p scattering, the electronic temperature $T_{\rm e}$ will be almost uniform along the device. Under this condition, $T_{\rm e}$ as a function of the Joule heating power P can be calculated from the nonlinear resistance according to the $R-T$ curve. $T_{\rm e}-P$ should be determined only by the e-p scattering and independent of the device resistance. Fig. S2b shows that $T_{\rm e}-P$ data for B=0, 1, 5 T collapse onto a single curve, providing strong evidence for a uniform $T_{\rm e}$, hence the e-p scattering being the dominant channel in heat dissipation.

The disordered graphene HEB is very sensitive. It shows response to RF noise at a few kelvin. The temperature dependence of the resistance is shown in Fig. S3 for two devices.

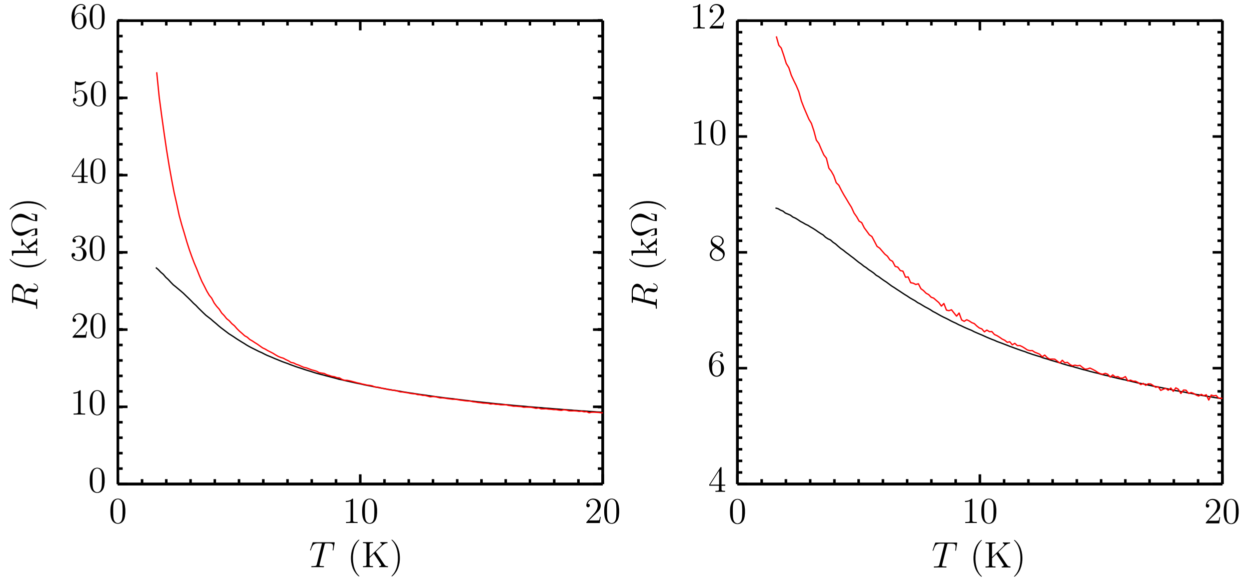

Fig. S3 Temperature dependence of resistance for two devices, (a) and (b), respectively. Black, data measured without a π filter at room temperature. Red, with a π filter.

The a.c. excitation is 0.5 nA. Two measurements were made for each device, with and without a room temperature π filter. Data from two measurements exhibit difference, which appears at low temperatures and becomes more significant as temperature decreases. Hot electrons created by RF noise heating usually emerge at very low temperature, *e.g.* a few hundred mK, as decoupling of electrons with phonons takes place at very low temperature for normal materials. However, the e-p coupling is very weak in graphene, the effect of RF noise heating can be seen at relatively high temperatures. It reflects the high sensitivity of disordered graphene HEB.

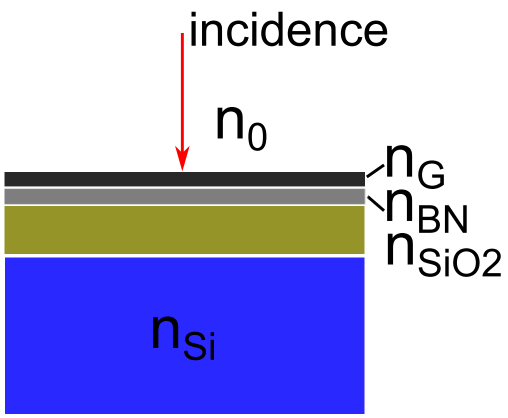

Fig. S4 Multilayer structure of disordered graphene bolometer.

To study the bolometric response of our HEB, a LED was placed over the device. It shines lights of wavelength peaking at 650 nm, 1.9 eV. The device absorbs a fraction of the incident light. To calculate the absorption, one has to consider the multilayer structure of the device, consisting of monolayer graphene, monolayer BN, 300 nm SiO_2 and a semi-infinite silicon substrate, schematically drawn in Fig. S4. In a multilayer structure, light will experience an infinite numbers of reflections and these reflections interfere[1]. Here, the optical transfer-matrix method is employed to conveniently calculate the absorptance[2]. At normal incidence, the characteristic matrix of a dielectric layer with a refractive index n can be written as,

$$\mathbf{M} = \begin{pmatrix} \cos\delta & i\sin\delta/n \\ in\sin\delta & \cos\delta \end{pmatrix} \tag{1}$$

where $\delta = 2nd/\lambda$. Here, d is the thickness of the layer and λ is the wavelength of light. For a multilayer, if the matrix for each layer is $\mathbf{M}_1, \mathbf{M}_2, ..., \mathbf{M}_n$, the matrix of the multilayer is $\mathbf{M} = \mathbf{M}_1 \cdot \mathbf{M}_2 \cdot ... \cdot \mathbf{M}_n$. The characteristic matrix of Graphene/BN/SiO_2 would be

$$\mathbf{M} = \mathbf{M}_{\mathrm{G}} \cdot \mathbf{M}_{\mathrm{BG}} \cdot \mathbf{M}_{\mathrm{SiO_2}} = \begin{pmatrix} m11 & m12 \\ m21 & m22 \end{pmatrix} \tag{2}$$

where $\mathbf{M}_{\mathrm{G}}, \mathbf{M}_{\mathrm{BG}}, \mathbf{M}_{\mathrm{SiO_2}}$ are the matrices of graphene, BN and SiO_2, respectively. The reflectance can be obtained by

$$R = |r|^2 = \left| \frac{n_0 m_{11} + n_0 n_3 m_{12} - m_{21} - n_3 m_{22}}{n_0 m_{11} + n_0 n_3 m_{12} + m_{21} + n_3 m_{22}} \right|^2 \tag{3}$$

, while the transmittance is

$$T = \frac{n_3}{n_0}|t|^2 = \frac{n_3}{n_0} \left| \frac{2n_0}{n_0 m_{11} + n_0 n_3 m_{12} + m_{21} + n_3 m_{22}} \right|^2 \tag{4}$$

Thus, the absorptance is

$$A = 1 - R - T \tag{5}$$

Monolayer graphene is 0.34 nm thick. Since the refractive index of disordered graphene is not known, we assume that it is the same as pristine graphene. For a wavelength of 650 nm, the refractive index n_{G} of pristine graphene is $2.6 - 1.3i$[1, 3]. For 0.333 nm BN, we take a refractive index $n_{\mathrm{BG}} = 1.7$. For SiO_2, $n_{\mathrm{SiO_2}} = 1.4563$, while for silicon, $n_{\mathrm{Si}} = 3.8392 + 0.0160i$[3]. Substitute these parameters in Eqs. 1-5, the absorptance of graphene can be computed, $A \approx 2.7\%$. We drive the LED by 1 mA current. Roughly

assuming an external quantum efficiency of 5% at 4 K[4, 5], the radiation power is $P_0 = 1.9$ V $\times 1$ mA $\times 5\% \approx 95$ μW. The LED has a view angle of 30° and is 2 mm away from the device. Knowing the active area of the device, 2.5×5 μm^2, the absorbed radiation is estimated as 40 pW.

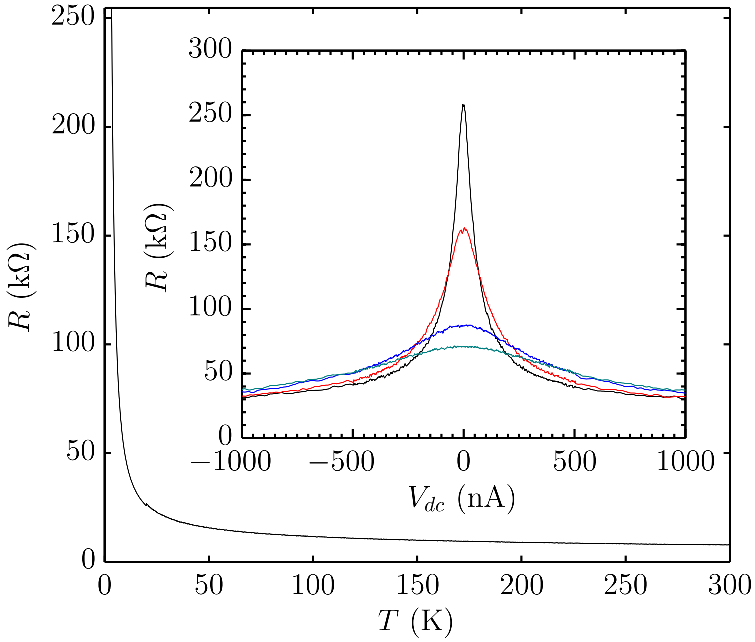

Fig. S5 Temperature dependence of the resistance for CVD graphene after bombardment by Ar plasma. Inset, the bias dependence of the resistance at 3, 4, 6, 7 K.

Devices made of disordered graphene film without Boron Nitride layer have also been studied. In fabrication, a CVD graphene film was first transferred to SiO_2/Si substrate and patterned into a graphene ribbon. Then, metal electrodes were deposited on the ribbon. The structure looks the same as those made of graphene/BN, except that there is no BN layer. To introduce disorder, devices were briefly, for $1 \sim 2$ s, exposed to Ar plasma. Raman spectra showed a strong D peak, signalling substantial disorder. The resistances of all devices show similar temperature and bias dependence. Fig. S5 shows the temperature dependence of the resistance for a device. The divergence of the resistance at low temperatures, manifest of localization, suggests a high responsivity for the bolometer. As seen in the inset of Fig. S5, the bias dependence of the resistance at a few temperatures exhibits the same nonlinearity

as in graphene/BN devices, indicating significant Joule heating.

---

[1] Blake, P. *et al.* Making graphene visible. *Appl. Phys. Lett.* **91**, 063124–3 (2007).

[2] Hecht, E. *Optics* (Addison-Wesley, 2001), 4th edn.

[3] Palik, E. D. *Handbook of Optical Constants of Solids* (Academic Press, New York, 1991).

[4] Li, Y. *et al.* Temperature dependence of the quantum efficiency in green light emitting diode dies. *Phys. Status Solidi (c)* **4**, 2784–2787 (2007).

[5] Temkin, H., Chin, A. K., DiGiuseppe, M. A. & Keramidas, V. G. Light-current characteristics of InGaAsP light emitting diodes. *Appl. Phys. Lett.* **39**, 405–407 (1981).
